# Assessing abdominal aortic aneurysm growth using radiomic features of perivascular adipose tissue after endovascular repair

**DOI:** 10.1186/s13244-024-01804-7

**Published:** 2024-09-30

**Authors:** Rui Lv, Ge Hu, Shenbo Zhang, Zhe Zhang, Jin Chen, Kefei Wang, Zhiwei Wang, Zhengyu Jin

**Affiliations:** 1 Department of Radiology, State Key Laboratory of Complex Severe and Rare Disease, Peking Union Medical College Hospital, Chinese Academy of Medical Sciences and Peking Union Medical College, Beijing, China; 2 Theranostics and Translational Research Center, National Infrastructures for Translational Medicine, Institute of Clinical Medicine, Peking Union Medical College Hospital, Chinese Academy of Medical Sciences and Peking Union Medical College, Beijing, China

**Keywords:** Abdominal aortic aneurysm, Endovascular aneurysm repair, Radiomics, Perivascular adipose tissue, Growth status classification

## Abstract

**Objectives:**

The study aimed to investigate the relationship between the radiomic features of perivascular adipose tissue (PVAT) and abdominal aortic aneurysm (AAA) growth after endovascular aneurysm repair (EVAR).

**Methods:**

Patients with sub-renal AAA who underwent regular follow-up after EVAR between March 2014 and March 2024 were retrospectively collected. Two radiologists segmented aneurysms and PVAT. Patients were categorised into growing and non-growing groups based on volumetric changes observed in two follow-up computed tomography examinations. One hundred seven radiomic features were automatically extracted from the PVAT region. Univariable and multivariable logistic regression was performed to analyse radiomic features and clinical characteristics. Furthermore, the performance of the integrated clinico-radiological model was compared with models using only radiomic features or clinical characteristics separately.

**Results:**

A total of 79 patients (68 ± 9 years, 89% men) were enroled in this study, 19 of whom had a growing aneurysm. Compared to the non-growing group, PVAT of growing AAA showed a higher surface area to volume ratio (non-growing vs growing, 0.63 vs 0.70, *p* = 0.04), and a trend of low dependence and high dispersion manifested by texture features (*p* < 0.05). The area under the curve of the integrated clinico-radiological model was 0.78 (95% confidence intervals 0.65–0.91), with a specificity of 87%. The integrated model outperformed models using only radiomic or clinical features separately (0.78 vs 0.69 vs 0.69).

**Conclusions:**

Higher surface area to volume ratio and more heterogeneous texture presentation of PVAT were associated with aneurysm dilation after EVAR. Radiomic features of PVAT have the potential to predict AAA progression.

**Clinical relevance statement:**

Radiomic features of PVAT are associated with AAA progression and can be an independent risk factor for aneurysm dilatation to assist clinicians in postoperative patient surveillance and management.

**Key Points:**

- After EVAR for AAA, patients require monitoring for progression.
- PVAT surrounding growing AAA after EVAR exhibits a more heterogeneous texture.
- Integrating PVAT-related features and clinical features results in better predictive performance.

**Graphical Abstract:**

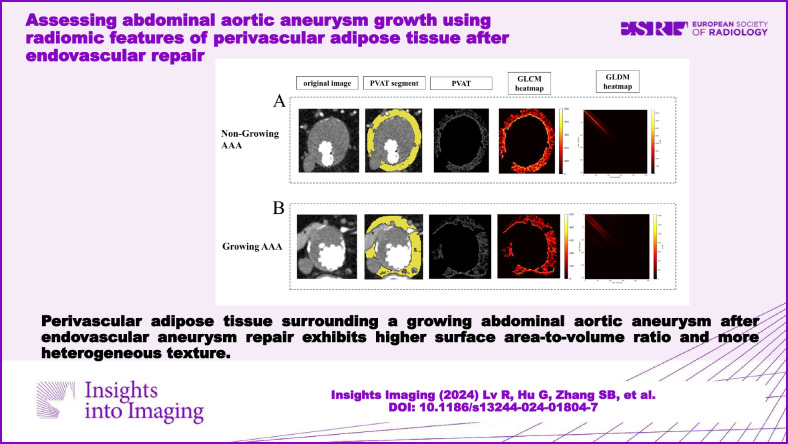

## Introduction

An abdominal aortic aneurysm (AAA) is defined as an aortic diameter of > 3 cm, with the primary risk being rupture and the associated risk of haemorrhagic death. Guidelines recommend repairing aneurysms with a maximum diameter exceeding 5.5 cm in males and 4.5 cm in females [1]. Endovascular aneurysm repair (EVAR) is a minimally invasive procedure that is safer than traditional open repair, but still requires long-term follow-up monitoring [2]. Therefore, exploring pertinent variables related to post-EVAR AAA growth is imperative for monitoring and treating AAA patients.

The causes of aneurysm growth are multifaceted, including endoleaks, endotension, hemodynamic factors, etc. Growing and non-growing groups could have factors contributing to aneurysm growth, and the level of these factors in the growing group was higher, whereas the non-growing group exhibited these factors to a lesser extent or not at all. Currently, research has shown an independent association between obesity and AAA development [3]. Perivascular adipose tissue (PVAT), which anatomically surrounds blood vessels, is now recognised as an active endocrine organ capable of secreting various adipokines, cytokines, and growth factors that can either hinder or promote the development of cardiovascular diseases [4, 5]. For aneurysms, adipose tissue infiltration into blood vessels and the secretion of proinflammatory factors may promote active macrophage infiltration, thereby inducing aneurysm development [6, 7].

Additionally, research has demonstrated a correlation between PVAT and the dimensions of the thoracic and abdominal aorta, suggesting that local fat deposits may contribute to aortic remodelling [8]. Furthermore, researchers have used the radiomic features of PVAT to assess coronary artery risk, yielding results that surpass traditional risk stratification methods [9]. For AAA, increased PVAT attenuation has been independently associated with AAA growth [10], and symptomatic aneurysms exhibit higher rates of adipose tissue attenuation [11]. However, studies have not focused on differences in the radiomic features of PVAT surrounding AAAs with different growth statuses. Moreover, they have not investigated whether radiomic features can be used to predict the classification of AAA growth status. Therefore, we aimed to investigate the relationship between the radiomic features of PVAT and the status of AAA growth after EVAR and to further explore the differentiation between growing and non-growing AAAs. We hypothesised that PVAT could help predict the status of AAA progression after EVAR.

## Materials and methods

### Study design and population

**Fig. 1:**
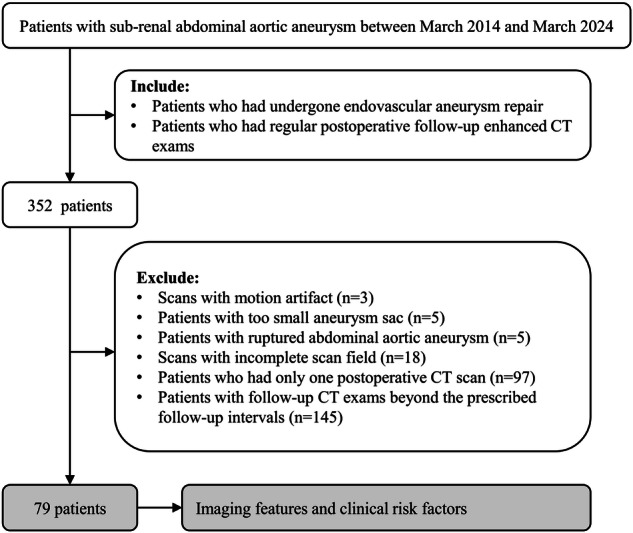
Patient selection flowchart

The Institutional Review Board of Peking Union Medical College Hospital approved this retrospective study and waived the requirement for informed consent. This retrospective study included all patients with sub-renal AAAs treated at Peking Union Medical College Hospital between March 2014 and March 2024. Patients were treated with aortic-covered stent grafts from Medtronic, Inc. (Endurant, Medtronic Inc). The inclusion criteria were endovascular repair of AAAs and regular postoperative follow-up with enhanced computed tomography (CT). The exclusion criteria included motion artefacts on scans (3 patients), small aneurysm sacs (5 patients), ruptured AAAs (5 patients), incomplete scan fields (18 patients), single follow-up CT (97 patients), and follow-up CT beyond the prescribed follow-up intervals (145 patients). The prescribed follow-up intervals were as follows: the first follow-up was scheduled 3–6 months after surgery, and the second follow-up was scheduled 9–12 months after the first follow-up. Figure 1 depicts the inclusion and exclusion criteria for patients. We retrieved clinical data from the electronic medical record system, including age, sex, history of cancer, hypertension, hyperlipidaemia, diabetes, coronary artery disease, peripheral artery disease, cerebral arteriopathy, smoking, drinking, low-density lipoprotein (LDL), high-density lipoprotein (HDL), triglycerides (TG), total cholesterol (TC), and glucose (GLU).

### Image segmentation

**Fig. 2:**
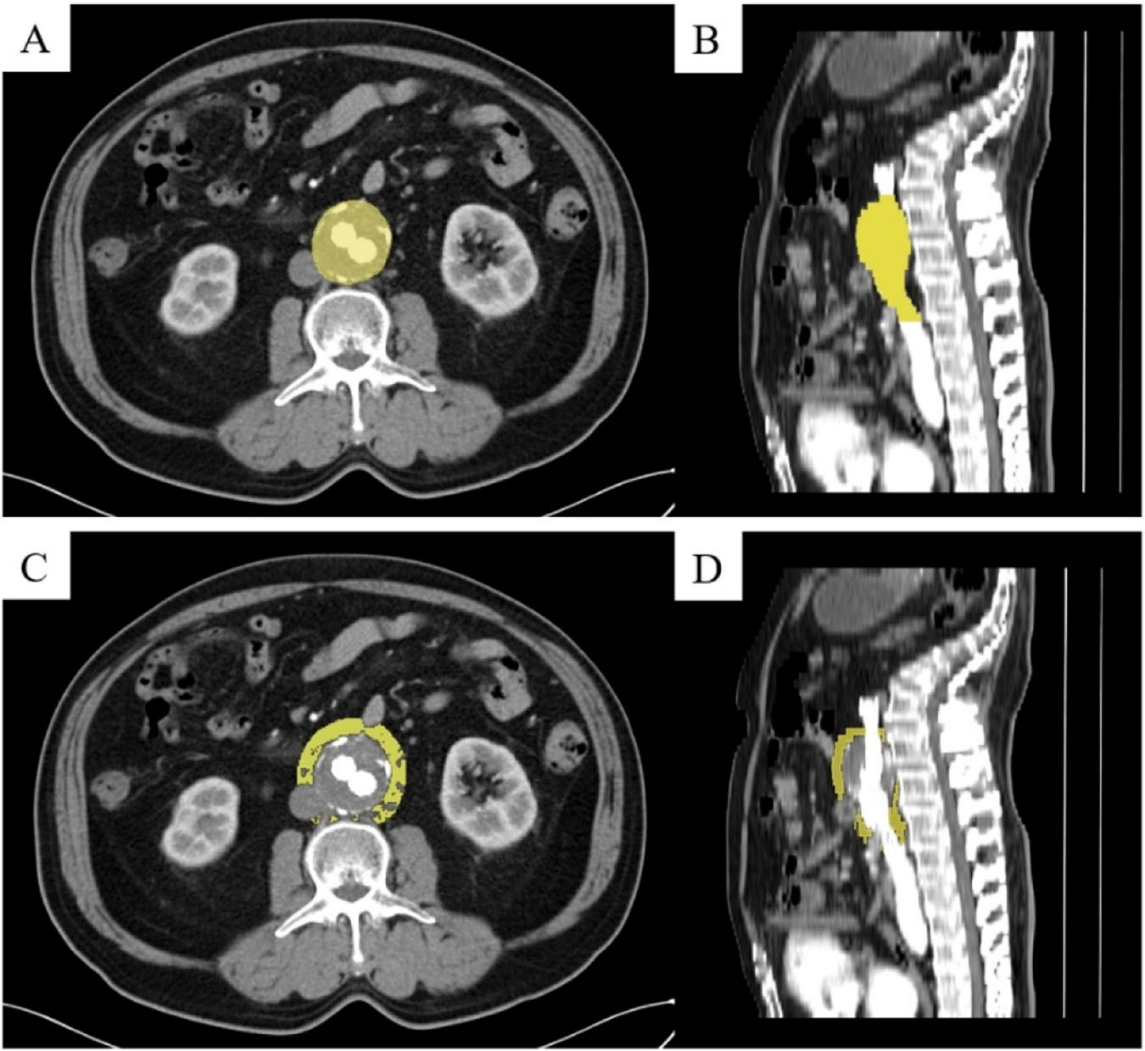
An example illustration of the segmentations of AAA and its PVAT. **A**, **B** A segmentation of AAA. **C**, **D** A segmentation of the PVAT of AAA. PVAT, perivascular adipose tissue; AAA, abdominal aortic aneurysm

Using the 3D Slicer software (version 5.0.3, https://www.slicer.org), two experienced radiologists, who have 5 years and 10 years of experience, respectively, manually created regions of interest (ROIs) on contrast-enhanced CT images to segment the three-dimensional AAA and calculate their volumes. The segmentation ranged from the lower renal artery level to the abdominal aorta bifurcation. We classified AAAs into growing and non-growing groups based on the volume change ratio of AAA. AAA was defined as growing if its volume increased by over 2% compared with its volume at the initial follow-up [12–15]. Subsequently, we utilised the “margin” function of the 3D Slicer software. This function grows or shrinks the selected segment by the specified margin. The segmentation of AAA was expanded outward by 10 mm [16, 17], and the original segmentation of the aneurysm was subtracted to obtain the segmentation of PVAT. The CT attenuation range was set to −195 to −45 [18, 19]. Figure 2 shows an example of the segmentation of AAA and its PVAT.

### Feature extraction

The radiomic features extracted from the PVAT region included 14 shape features, 18 histogram features (also known as first-order features), and 75 texture features. The histogram features describe the statistical distribution of CT attenuation within the ROI [20]. Texture features quantify the relationships between voxel intensities and their surroundings [21], including grey-level co-occurrence matrix (GLCM) features, grey-level dependence matrix (GLDM) features, grey-level run length matrix (GLRLM) features, grey-level size zone matrix (GLSZM) features, and neighbouring grey tone difference matrix (NGTDM) features. Detailed descriptions of the radiomic features can be found in supplementary materials and a separate document by Zwanenburg et al [22]. During image segmentation and feature extraction, the researchers were blinded to the patient’s clinical information, including group classifications.

### Statistical analysis

Statistical analyses were conducted using SPSS (version 27.0; International Business Machines Corporation). Following tests for variance homogeneity and normal distribution of variables, we conducted univariate analysis of the variables using independent samples *t*-test, non-parametric Mann–Whitney *U*-test, χ2 test, or Fisher’s exact test, depending on the type of variable. To address multicollinearity among variables, we calculated the correlations between them. A correlation coefficient of > 0.8 was considered indicative of collinearity. One of the two highly correlated variables was removed, while retaining the one with the highest correlation with the dependent variable. This approach allowed us to select the most relevant independent variables for further analysis. Variables exhibiting statistically significant differences in group comparisons were included in the binary logistic regression analysis to identify independent risk factors for AAA growth. Odds ratios (ORs) and their respective 95% confidence intervals (CIs) were determined. Receiver operating characteristic (ROC) curves were constructed to evaluate the predictive performance of the identified factors, and the area under the curve (AUC) was calculated. Differences were considered statistically significant at *p* < 0.05 (two-tailed).

## Results

The agreement between the two radiologists regarding AAA growth classification was excellent (Cohen’s kappa coefficient, 0.97; 95% CI: 0.75–1.19; *p* < 0.001).

### Enrolment of participants

We conducted sample size estimation during the pre-experiment phase with a significance level (α) of 0.05 (two-tailed) and power (1-β) of 0.90. Consequently, a sample size of 78 patients was obtained. Between March 2014 and March 2024, 352 consecutive patients underwent endovascular repair for AAAs and regular postoperative follow-up enhanced CT. Ultimately, 79 patients were included in this study. Table 1 summarises the detailed demographic and clinical characteristics.

**Table 1:** Demographic and clinical characteristics of participants

Variables	Overall, (*n* = 79)		*p*-value
	Non-growing AAA, (*n* = 60)	Growing AAA, (*n* = 19)	
Age, year	68 ± 8	69 ± 11	0.70
Male gender, *n* (%)	54 (90%)	16 (84%)	0.47
History of tumour, *n* (%)	10 (17%)	2 (11%)	0.72
History of hypertension, *n* (%)	38 (63%)	9 (47%)	0.22
History of hyperlipemia, *n* (%)	16 (27%)	5 (26%)	0.98
History of diabetes, *n* (%)	10 (17%)	0 (0%)	0.11
History of coronary artery disease, *n* (%)	14 (23%)	5 (26%)	0.77
History of peripheral arterial disease, *n* (%)	11 (18%)	3 (16%)	> 0.99
History of cerebral arteriopathy, *n* (%)	10 (17%)	1 (5%)	0.28
History of smoking, *n* (%)	36 (60%)	9 (47%)	0.33
History of drinking, *n* (%)	18 (30%)	4 (21%)	0.56
LDL, mmol/L	2.46 ± 0.72	3.03 ± 1.17	0.01
HDL, mmol/L	0.93 ± 0.18	1.08 ± 0.35	0.02
TG, mmol/L	1.54 ± 0.78	1.57 ± 0.85	0.89
TC, mmol/L	4.10 ± 0.82	4.87 ± 1.32	< 0.01
GLU, mmol/L	5.53 ± 1.14	5.38 ± 1.04	0.61

The data were presented as mean ± standard deviation or as numbers (percentages)

*AAA* abdominal aortic aneurysm, *LDL* low-density lipoprotein, *HDL* high-density lipoprotein, *TG* triglycerides, *TC* total cholesterol, *GLU* glucose

### Univariate analysis among non-growing and growing AAA after EVAR

Table 1 presents the statistical data on the clinical characteristics of patients with growing or non-growing AAA after EVAR. LDL (2.46 vs 3.03, *p* = 0.01), HDL (0.93 vs 1.08, *p* = 0.02), and TC (4.10 vs 4.87, *p* = 0.02) showed significant differences between the growing or non-growing AAA groups, whereas no significant differences were found in the remaining clinical parameters.

Table 2 summarises the statistical data of the radiomics features of the PVAT surrounding growing or non-growing AAAs after EVAR. It comprises 1 shape feature and 13 texture features, including GLCM, GLDM, and GLRLM features. Significant differences between the two groups were observed, including surface area-to-volume ratio (0.63 vs 0.70, *p* = 0.04), correlation (0.53 vs 0.50, *p* = 0.04), an informational measure of correlation 1 (0.14 vs 0.13, *p* = 0.03), Inverse variance (0.46 vs 0.47, *p* = 0.04), maximal correlation coefficient (0.63 vs 0.57, *p* = 0.04), Maximum probability (0.22 vs 0.20, *p* = 0.03), dependence non-uniformity normalised (0.05 vs 0.06, *p* = 0.02), Dependence variance (29.88 vs 26.96, *p* = 0.01), large dependence emphasis (141.17 vs 124.55, *p* = 0.02), long-run emphasis (4.76 vs 4.20, *p* = 0.01), run length non-uniformity normalised (0.50 vs 0.52, *p* = 0.01), run percentage (0.64 vs 0.66, *p* = 0.01), run variance (1.80 vs 1.50, *p* = 0.01), and short-run emphasis (0.72 vs 0.74, *p* = 0.04). Regarding histogram features, we did not find any evidence of differences between the two groups. Regarding texture features, including GLRLM, GLSZM, and NGTDM features, we also did not find statistically significant results. Table 2 shows the detailed results.

**Table 2:** Univariate analysis of radiomics features of PVAT among non-growing and growing AAA

Variables	Non-growing AAA	Growing AAA	*p*-value
Shape features			
Surface area to volume ratio	0.63 ± 0.14	0.70 ± 0.13	0.04
Grey-level co-occurrence matrix features			
Correlation	0.53 ± 0.07	0.50 ± 0.05	0.04
Informational measure of correlation 1	0.14 ± 0.03	0.13 ± 0.02	0.03
Inverse variance	0.46 ± 0.02	0.47 ± 0.02	0.04
Maximal correlation coefficient	0.63 ± 0.11	0.57 ± 0.08	0.04
Maximum probability	0.22 ± 0.04	0.20 ± 0.04	0.03
Grey-level dependence matrix features			
Dependence non-uniformity normalised	0.05 ± 0.01	0.06 ± 0.005	0.02
Dependence variance	29.88 ± 4.51	26.96 ± 4.13	0.01
Large dependence emphasis	141.17 ± 27.97	124.55 ± 22.08	0.02
Grey-level run length matrix features			
Long run emphasis	4.76 ± 1.00	4.20 ± 0.78	0.01
Run length non-uniformity normalised	0.50 ± 0.05	0.52 ± 0.04	0.01
Run percentage	0.64 ± 0.04	0.66 ± 0.03	0.01
Run variance	1.80 ± 0.53	1.50 ± 0.42	0.01
Short run emphasis	0.72 ± 0.04	0.74 ± 0.03	0.04

*PVAT* perivascular adipose tissue, *AAA* abdominal aortic aneurysm

**Fig. 3:**
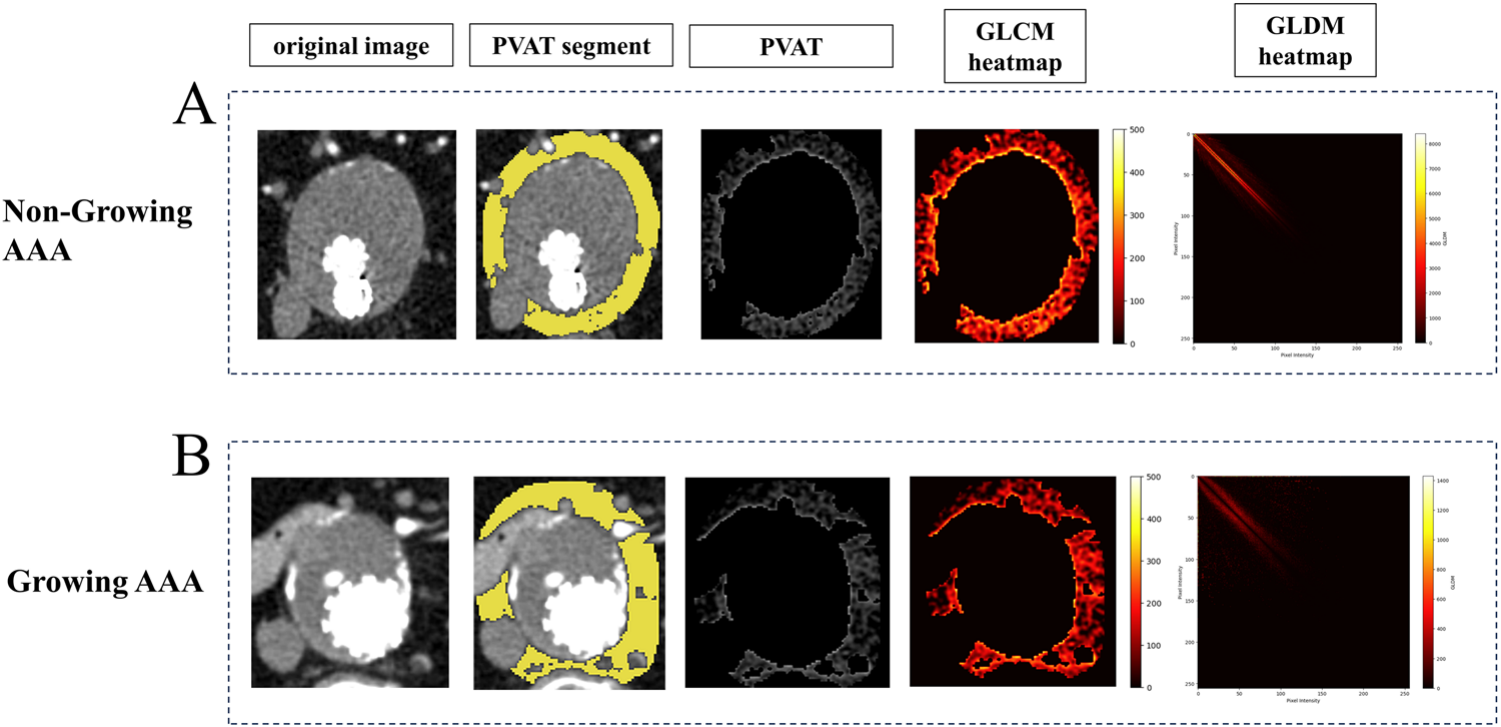
The visualisation of texture features. **A** A 68-year-old male in the non-growing AAA group. The first image in the top row displays the arterial phase enhanced CT slice of the maximum axial plane of the AAA. The second image from the top represents the manual segmentation of PVAT conducted by radiologists. The third image depicts the extracted PVAT. The fourth and fifth images display the heatmaps of the GLCM and GLDM, respectively. **B** A 74-year-old male in the growing AAA group. The image of (**B**) represents the same meaning as (**A**). AAA, abdominal aortic aneurysm; PVAT, perivascular adipose tissue; GLCM, grey level co-occurrence matrix; GLDM, grey level dependence matrix

Figure 3 illustrates two examples of the texture of PVAT surrounding growing or non-growing AAAs. Comparing the GLCM texture heatmaps of PVAT, growing AAAs exhibited darker regions with lower GLCM values, whereas non-growing AAAs exhibited brighter regions with higher GLCM values. The GLDM texture heatmaps showed that growing AAAs exhibited patterns farther from the diagonal lines. In Fig. 3, differences in the texture of PVAT were visually observed between the two groups.

### Binary logistic regression analysis among non-growing and growing AAA after EVAR

Among the 79 patients, 19 (24%) had non-growing AAA. The variables included in the binary logistic regression model were determined using univariate analysis (*p* = 0.05). The dependence variances of PVAT and TC were included in the binary logistic regression model.

**Fig. 4:**
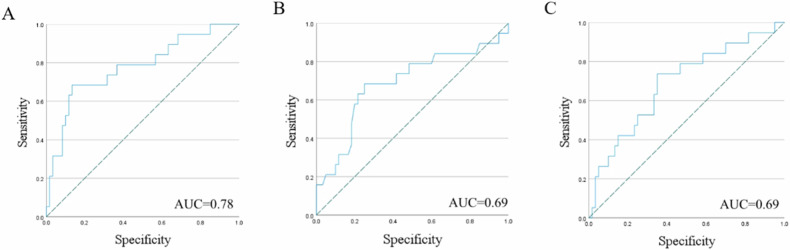
ROC curves of radiomic signature in predicting AAA growth classification in different models. **A** Clinico-radiological integrated model. **B** Radiomic model. **C** Clinical model. AAA, abdominal aortic aneurysm; AUC, area under the curve

The ORs for these two features are 0.84 (95% CI: 0.72–0.98, *p* = 0.03) and 2.24 (95% CI 1.20–4.17, *p* = 0.01), respectively. The AUC of this model was 0.78 (95% CI: 0.65–0.91, *p* < 0.01), with a sensitivity of 68% and specificity of 87%. Additionally, by comparing the model combining radiomic and clinical features with models using only radiomic features or clinical features separately, their AUCs were 0.69 (95% CI: 0.55–0.83, *p* = 0.02) and 0.69 (95% CI: 0.54–0.84, *p* = 0.02), respectively. Table 3 lists the evaluation indices of the three models. All three models showed statistical significance (Omnibus test, *p* < 0.01) and had good goodness-of-fit (Hosmer–Lemeshow test, *p* > 0.05). We found that the clinico-radiological integrated model outperformed the individual models. Figure 4 shows the ROC curves of the three models.

**Table 3:** Evaluation of logistic regression model for AAA growth

Evaluation index	Radiomic model	Clinical model	Clinico-radiological integrated model
The *p*-value of omnibus test	0.01	< 0.01	< 0.01
The *p*-value of Hosmer–Lemeshow test	0.98	0.24	0.47
Percentage accuracy in classification (%)	75%	79%	80%
AUC (95% CI)	0.69 (0.55–0.83)	0.69 (0.54–0.84)	0.78 (0.65–0.91)
The *p*-value of AUC	0.02	0.02	< 0.01
Sensitivity	74%	74%	68%
Specificity	65%	65%	87%
Cutoff value	0.25	0.25	0.31

*AAA* abdominal aortic aneurysm, *AUC* area under curve, *CI* confidence interval

## Discussion

In this study, we analysed the radiomic features of PVAT on follow-up enhanced CT after EVAR and the clinical characteristics of patients to explore the association between PVAT and AAA growth status. The clinical-radiological integrated model achieved the best performance, with an AUC of 0.78. Our finding holds significant clinical value for the long-term follow-up of patients after EVAR.

The results of the univariate analysis indicated that PVAT surrounding growing AAA after EVAR had a higher surface area-to-volume ratio, which aligns with the theory of fat adhering closely to blood vessels and potentially facilitating the infiltration or secretion of PVAT [4, 23]. Regarding texture features, previous studies have shown that texture analysis of aneurysms and thrombi can effectively predict aneurysm expansion [12, 24]. In our study, both GLCM and GLDM results indicated that PVAT surrounding growing AAA exhibited low dependence and high dispersion. The heatmaps of GLCM and GLDM for growing and non-growing AAAs illustrate texture differences between the two, suggesting that the internal composition of PVAT in growing AAAs is more heterogeneous [22]. This discovery may motivate researchers to investigate the use of PVAT texture features in classifying AAA growth.

Dependence variance describes the texture and structure of images and measures the variability of the greyscale differences between pairs of pixels across the entire image. The calculation is done using GLCM, where the variance of greyscale differences in GLCM yields dependence variance [20, 21]. Our results indicate that a lower variability in greyscale differences of PVAT, indicating more uniform greyscale differences, may correspond to a higher risk of AAA growth after EVAR. Serum TC measurement includes various types of cholesterol particles present in the blood. Studies have shown a positive correlation between serum TC levels and AAA growth [25, 26], which is consistent with previous findings.

No significant difference was found in CT attenuation between the two PVAT groups (−81.80 vs −80.06, *p* = 0.28), with a single-factor logistic analysis yielding an AUC value of 0.596 (95% CI: 0.448–0.744, *p* = 0.21), which was significantly lower than that of the other models in this study. This may benefit future research in identifying the relevant features for predicting AAA expansion, especially in post-EVAR studies.

Long-term follow-up is required to monitor evolutions after EVAR [2]. Some researchers have focused on monitoring postoperative complications, such as persistent type 2 endoleaks, and radiomics features have been used to establish machine learning algorithms for predicting aggressive type 2 endoleaks after EVAR [27]. Researchers have also developed radiomics models for AAA to predict the outcomes of various postoperative complications after EVAR [28, 29]. Rapid growth in volume or diameter typically indicates unfavourable evolution, and monitoring these parameters may facilitate the early detection of AAA evolutions. For instance, efforts have been made to identify the post-EVAR shrinkage of AAA [30] or to classify post-EVAR AAA evolutions using radiomics texture analysis [31]. This study monitored changes in AAA volume and demonstrated that differences in PVAT may lead to variations in AAA volume.

PVAT has been extensively studied for its pathophysiological effects on blood vessels [4, 23, 32]. Due to its significant impact on blood vessels, researchers have attempted to integrate PVAT with traditional predictive features in previous studies. For example, adding PVAT radiomics to the CT-derived fractional flow reserve model enhanced the diagnostic performance for detecting haemodynamically significant coronary artery stenosis [33]. Extracting the radiomics features of PVAT and carotid plaques improved the model’s capability to identify symptomatic carotid plaques [34]. Current research on PVAT of AAA has often focused on the attenuation of adipose tissue around the aorta [10, 16, 35], highlighting its association with AAA growth. Additionally, studies have shown that PVAT density around the aneurysm sac exceeds that in healthy vessels [11]. Our study expands the scope of the application of PVAT in predicting AAA growth by demonstrating the relationship between the radiomics features of PVAT and post-EVAR AAA evolution. Integrating these features into predictive models, alongside traditional clinical features, improves the model’s predictive performance. Multivariate Cox regression analysis revealed that the baseline maximum diameter and high PVAT attenuation were independent predictors of AAA progression [10]. Furthermore, one study found that the average CT attenuation of PVAT predicted AAA growth with an AUC of 0.688, which increased to 0.797 when combined with the baseline AAA diameter [16]. Our model demonstrated a similar performance to theirs. Furthermore, the model achieved a specificity of 87%, indicating its ability to effectively exclude false positives and minimise the misclassification of non-growing AAA cases as growth cases. In future research, we plan to establish a comprehensive model that incorporates the aneurysm sac, intraluminal thrombus, clinical features, and PVAT to evaluate the predictive value of PVAT and the predictive ability of the model.

This study has several limitations. Due to the need for relatively stable conclusions, strict follow-up intervals were set, resulting in a smaller final sample size. We calculated the estimated sample size for the overall study in the pre-experiment to minimise the impact of the small sample size. In the future, we will increase the sample size to further explore the relationship between PVAT and AAA. This retrospective study highlighted the necessity for prospective and long-term follow-up studies. There were also limitations related to the development of image segmentation techniques, necessitating manual intervention for three-dimensional aneurysm segmentation. Currently, complete automation through software programmes remains unattainable.

In conclusion, the PVAT of growing AAA after EVAR exhibits a higher surface area-to-volume ratio and more heterogeneous texture. The radiomic features of PVAT exhibit significant differences between patients with growing or non-growing AAA and can help categorise the AAA growth status after EVAR with high specificity. Integrating PVAT-related features into predictive models alongside traditional clinical features can improve the model’s predictive performance.

## Supplementary information

ELECTRONIC SUPPLEMENTARY MATERIAL

## Data Availability

The datasets generated and/or analysed during the current study are available from the corresponding author upon reasonable request.

## Abbreviations

AAA: Abdominal aortic aneurysm
AUC: Area under curve
CI: Confidence interval
CT: Computed tomography
EVAR: Endovascular aneurysm repair
GLCM: Grey-level co-occurrence matrix
GLDM: Grey-level dependence matrix
GLRLM: Grey-level run length matrix
GLSZM: Grey-level size zone matrix
GLU: Glucose
HDL: High-density lipoprotein
LDL: Low-density lipoprotein
NGTDM: Neighbouring grey tone difference matrix
OR: Odds ratio
PVAT: Perivascular adipose tissue
ROC: Receiver operating characteristic
ROI: Region of interest
TC: Total cholesterol
TG: Triglycerides
