# Supplementary material for: Assessing abdominal aortic aneurysm growth using radiomic features of perivascular adipose tissue after endovascular repair
Source: Insights Imaging. 2024 Sep 30;15:232. doi: 10.1186/s13244-024-01804-7 (PMC11442904; doi:10.1186/s13244-024-01804-7)
Supplement: Supplementary file 1 — ELECTRONIC SUPPLEMENTARY MATERIAL [file 13244_2024_1804_MOESM1_ESM.pdf]

Assessing abdominal aortic aneurysm growth using radiomic features of perivascular adipose tissue after endovascular repair

ELECTRONIC SUPPLEMENTARY MATERIAL CT protocol

All enrolled patients had undergone contrast-enhanced CT examination, primarily using a dual-source CT scanner (SOMATOM Definition Flash, Siemens Healthcare). The imaging protocol was biphasic, with the patient in a supine position, scanning from the level of the diaphragm to the level of the symphysis pubis. Following the acquisition of unenhanced CT images, contrast agent (370 mgI/mL, Iopromide, Ultravist, Bayer Healthcare) was injected through the median right elbow at a rate of 4 mL/s. A bolus tracking technique (trigger threshold 100 HU; trigger level, abdominal aorta) was used to trigger the acquisition of contrast-enhanced CT scans. All acquired images were reconstructed according to the DICOM (Digital Imaging and Communications in Medicine) standard into images with a slice thickness of 1 mm (for image evaluation) and 5 mm (for image segmentation), with a size of 512×512 pixels per CT slice.

Radiomic features

a) Histogram features, which are also called first-order features, describe the intensity and distribution of CT attenuation values within the interested region (the perivascular adipose tissue of abdominal aortic aneurysm) defined by the mask through commonly used and basic metrics, including the following 18 statistics.

- 10th Percentile: the 10th percentile CT attenuation within the region of interest (ROI).
- 90th Percentile: the 90th percentile CT attenuation within the ROI.
- Energy: energy is a measure of the magnitude of voxel values in a CT image. A larger value implies a greater sum of the squares of these values.
- Entropy: entropy specifies the uncertainty/randomness in the CT attenuation. It measures the average amount of information required to encode the CT values.
- Interquartile Range: the CT values between the 25th and 75th percentile.
- Kurtosis: kurtosis is a measure of the peakedness of the distribution of CT attenuation in the ROI. A higher kurtosis implies that the mass of the distribution is concentrated towards the tail(s) rather than towards

the mean. A lower kurtosis implies the reverse: that the mass of the distribution is concentrated towards a spike near the Mean value.

- Maximum: the maximum CT attenuation within the ROI.
- Mean: the average CT value within the ROI.
- Mean absolute deviation (MAD): MAD is the mean distance of all CT values from the mean value of the CT image array.
- Median: the median CT value within the ROI.
- Minimum: the minimum CT attenuation within the ROI.
- Range: the range of CT values in the ROI.
- Robust mean absolute deviation (RMAD): RMAD is the mean distance of all CT values from the Mean Value calculated on the subset of image array with CT values in between, or equal to the 10th and 90th percentile.
- Root mean squared (RMS): RMS is the square-root of the mean of all the squared CT attenuation. It is another measure of the magnitude of the image values.
- Skewness: skewness measures the asymmetry of the distribution of CT attenuation about the mean value. Depending on where the tail is elongated and the mass of the distribution is concentrated, this value can be positive or negative.
- Total Energy: Total Energy is the value of Energy feature scaled by the volume of the voxel in cubic mm.
- Uniformity: uniformity is a measure of the sum of the squares of each CT attenuation. This is a measure of the homogeneity of the image array, where a greater uniformity implies a greater homogeneity or a smaller range of discrete CT values.
- Variance: Variance is the mean of the squared distances of each CT attenuation from the mean value. This is a measure of the spread of the distribution about the mean.

b) Shape Features

- Elongation: Elongation shows the relationship between the two largest principal components in the ROI shape. For computational reasons, this feature is defined as the inverse of true elongation.
- Flatness: Flatness shows the relationship between the largest and smallest principal components in the ROI shape. For computational reasons, this feature is defined as the inverse of true flatness.
- Least Axis Length: The principal component analysis is performed using the physical coordinates of the voxel centers defining the ROI. It therefore takes spacing into account, but does not make use of the shape mesh.
- Major Axis Length: The principal component analysis is performed using the physical coordinates of the voxel centers defining the ROI. It therefore takes spacing into account, but does not make use of the shape mesh.

- Maximum 2D diameter (Column): Maximum 2D diameter (Column) is defined as the largest pairwise Euclidean distance between tumor surface mesh vertices in the row-slice (usually the coronal) plane.
- Maximum 2D diameter (Row): Maximum 2D diameter (Row) is defined as the largest pairwise Euclidean distance between tumor surface mesh vertices in the column-slice (usually the sagittal) plane.
- Maximum 2D diameter (Slice): Maximum 2D diameter (Slice) is defined as the largest pairwise Euclidean distance between tumor surface mesh vertices in the row-column (generally the axial) plane.
- Maximum 3D diameter: Maximum 3D diameter is defined as the largest pairwise Euclidean distance between tumor surface mesh vertices.Also known as Feret Diameter.
- Mesh Volume: Calculate the volume through the triangular grid of ROI.
- Minor Axis Length: The principal component analysis is performed using the physical coordinates of the voxel centers defining the ROI. It therefore takes spacing into account, but does not make use of the shape mesh.
- Sphericity: Sphericity is a measure of the roundness of the shape of the tumor region relative to a sphere. It is a dimensionless measure, independent of scale and orientation.
- Surface Area: the extent of a 2D surface enclosed within a boundary
- Surface Area to Volume ratio: This feature is not dimensionless, and is therefore (partly) dependent on the volume of the ROI.
- Voxel Volume: This is a less precise approximation of the volume and is not used in subsequent features. This feature does not make use of the mesh and is not used in calculation of other shape features.

c) Texture features quantify the relationship between voxels and their surroundings of both distance and intensity, containing 75 statistics in the following 5 categories.

- Gray level co-occurrence matrix (GLCM) features (24 features): GLCM describes the second-order joint probability function of an image region constrained by the matrix that computes how often pairs of pixels with a specific value and offset occur in the image.
- Gray level dependence matrix (GLDM) features (14 features): GLDM quantifies gray level dependencies in an image. A gray level dependency is defined as the number of connected voxels within distance δ that are dependent on the center voxel.
- Gray level run length matrix (GLRLM) features (16 features): GLRLM quantifies gray level runs, which are defined as the length in number of pixels, of consecutive pixels that have the same CT value.
- Gray level size zone matrix (GLSZM) features (16 features): GLSZM quantifies CT attenuation zones in an image. A CT attenuation zone is defined as the number of connected voxels that share the same gray level intensity. A voxel is considered connected if the distance is 1

according to the infinity norm.

- Neighboring gray tone difference matrix (NGTDM) features (5 features): NGTDM quantifies the difference between a CT value and the average CT value of its neighbors within distance δ.
